# Therapeutic Applications of Ginseng Natural Compounds for Health Management

**DOI:** 10.3390/ijms242417290

**Published:** 2023-12-09

**Authors:** Syed Sayeed Ahmad, Khurshid Ahmad, Ye Chan Hwang, Eun Ju Lee, Inho Choi

**Affiliations:** 1 Department of Medical Biotechnology, Yeungnam University, Gyeongsan 38541, Republic of Korea; sayeedahmad4@gmail.com (S.S.A.); ahmadkhursheed2008@gmail.com (K.A.); 1230hyc@naver.com (Y.C.H.); gorapadoc0315@hanmail.net (E.J.L.); 2 Research Institute of Cell Culture, Yeungnam University, Gyeongsan 38541, Republic of Korea

**Keywords:** ginseng, compounds, treatment, disease, mechanism

## Abstract

Ginseng is usually consumed as a daily food supplement to improve health and has been shown to benefit skeletal muscle, improve glucose metabolism, and ameliorate muscle-wasting conditions, cardiovascular diseases, stroke, and the effects of aging and cancers. Ginseng has also been reported to help maintain bone strength and liver (digestion, metabolism, detoxification, and protein synthesis) and kidney functions. In addition, ginseng is often used to treat age-associated neurodegenerative disorders, and ginseng and ginseng-derived natural products are popular natural remedies for diseases such as diabetes, obesity, oxidative stress, and inflammation, as well as fungal, bacterial, and viral infections. Ginseng is a well-known herbal medication, known to alleviate the actions of several cytokines. The article concludes with future directions and significant application of ginseng compounds for researchers in understanding the promising role of ginseng in the treatment of several diseases. Overall, this study was undertaken to highlight the broad-spectrum therapeutic applications of ginseng compounds for health management.

## 1. Introduction

Natural products are viewed as a primary source of therapeutic agents and have been identified in plants, microorganisms, animals, insects, and marine organisms [1]. Natural products have diverse pharmacological properties and play important roles in drug discovery and development by serving as novel lead templates. Aspirin (from willow tree bark), digoxin (from the flower; *Digitalis lanata*), morphine (from opium), artemisinin, camptothecin, lovastatin, maytansine, reserpine, and silibinin are just a few examples of drugs directly or indirectly derived from natural products [2]. Some semi-synthetic therapeutic agents (hybrids of natural and synthetic sources), such as penicillin [3] and paclitaxel (an anti-cancer drug derived from the Pacific yew, *Taxus brevifolia*) [4], are typically produced by chemically transforming natural products [5]. The chemical, functional, and structural diversities of small molecule natural products have been explored [2]. The interactions between biological macromolecules (mainly proteins) and natural products explain the therapeutic efficacies of natural products. Furthermore, natural products do not cause as many adverse effects as synthetic compounds and combinatorial libraries [2].

The use of Chinese medicine has risen in popularity after the 2015 Nobel Prize was awarded for the discovery that artemisinin is an effective treatment for malaria [6]. Ginseng (a medicinal herb) and its derived natural products are amongst the most popular natural remedies and are used to treat various diseases and conditions such as diabetes [7], anti-oxidative [8], inflammation [9], cancers [10], fungal, bacterial, viral, stress [11], and neurodegenerative diseases (ND) [12], as well as brain ischemia [13], hypertension [14], obesity [15], cardiovascular diseases and stroke [16], sarcopenia [17], muscle-wasting conditions [18,19,20], muscle aging, and cancer cachexia [21,22,23]. Known side effects of ginseng include headaches, diarrhea, blood pressure changes, skin irritations, and vaginal bleeding [24]. Overall, ginseng has been reported to be a useful management option for many diseases, as is suggested by its name—Panax is derived from the Greek pan akheia, meaning “cures all diseases” [25]. A summary of known ginseng compounds is provided in Table 1.

Seventeen Panax species are now recognized, but commercial *P. ginseng* cultivars are largely found in South Korea and China [26]. The two most well-known are *Panax ginseng* and *Panax quinquefolius* [27]. The world’s major ginseng producers are China, South Korea, Canada, and the United States [27], and South Korea is the largest ginseng distributor [27]. *P. ginseng* and its ginsenoside components are non-toxic and used to treat chemotherapy-induced side effects such as nephrotoxicity, hepatotoxicity, cardiotoxicity, immunotoxicity, and hematopoietic suppression [28]. Here, we report the broad-spectrum therapeutic applications of known ginseng compounds. The strategy used to identify the articles include performing thorough searches in reputable academic databases such as PubMed, Scopus, SciFinder, Science Direct, Google Scholar, and the Scientific Information Database. The study focused on English language papers with particular keywords related to ginseng plants, natural compounds, biological investigations, and activities. The aim of this review was to explore ginseng-related natural compounds with potential use for the management of human health.

## 2. Protopanaxadiol (PPD) and Protopanaxatriol (PPT)

Protopanaxadiol (PPD) and protopanaxatriol (PPT) are active compounds found in members of the *Panax* genus, mainly in the roots, stems, leaves, and flowers. PPD is used to treat endometriosis and has been shown to significantly upregulate endometrial receptivity-related molecules, such as interleukin 6 family cytokine, insulin-like growth factor-binding protein 1, and collagens, to restrict the pelvic macrophage inflammatory response and to recover fertility in mice with endometriosis. Thus, the literature shows that PPD prevents and is a promising treatment for endometriosis [29]. On the other hand, PPT ginsenosides have pharmacological effects on the central nervous and cardiovascular systems [30], and PPT reportedly acts as a PPARγ antagonist [31]; thus, targeting PPARγ is considered a promising treatment option for obesity.

## 3. Ginsenoside F1 (GF1)

GF1 is a ginseng saponin isolated from a traditional Chinese medicine used to treat ischemic stroke. GF1 activates the IGF-1/IGF1R pathway [32] to promote angiogenesis, which reduces cerebral ischemia. In addition, GF1 may improve cerebrovascular function and accelerate recovery from ischemic stroke. In zebrafish, GF1 repaired vascular defects caused by axitinib [33], and in vivo and in vitro studies revealed that GF1 protects against Aβ accumulation. At 2.5 μM, GF1 reduced Aβ-induced cytotoxicity by reducing Aβ accumulation in mouse neuroblastoma neuro-2a (N2a) and human neuroblastoma SH-SY5Y neuronal cell lines. Additionally, GF1 reduced Aβ plaques in the hippocampus of (APP/PS1) double-transgenic AD mice [34,35]. Collectively, studies have shown GF1 is a highly active component in *P. ginseng* that can cross the BBB and has therapeutic potential for treating ND. Furthermore, GF1 reduces eosinophilic inflammation in chronic rhinosinusitis by enhancing NK cell activity [36].

## 4. Ginsenoside F2 (GF2)

GF2 is a minor component in *P. ginseng* with therapeutic applications in inflammatory diseases [37]. GF2 treatment attenuated liver damage in C57BL/6J WT mice (a model of alcoholic liver injury) [37] and suppressed the expression of TGF-β2 (a pro-apoptotic factor) to reduce hair loss in a dihydrotestosterone-induced mouse model [38]. It has been well established that excessive alcohol consumption can result in vitamin/mineral shortages (possibly due to liver damage) and subsequent hair loss because appropriate nourishment is required to maintain hair quality. In addition, alcohol suppresses nutrient breakdown and the body’s capacity to absorb nutrients. Currently, hair loss is a major issue among young men. We suggest that the effects of GF2 on known targets of hair loss should be investigated to manage this condition.

## 5. Ginsenoside F3 (GF3)

GF3 was isolated from the leaves of *P. ginseng* [39]. At concentrations ranging from 0.1 to 100 μmol/L, GF3 not only stimulated murine spleen cell proliferation but also raised the production of IL-2 and IFN-γ. It improved immunity by modulating the synthesis and gene expression of type 1 and 2 cytokines in murine spleen cells [40].

## 6. Ginsenoside F4 (GF4)

GF4 considerably enhanced the hyperglycemic state of db/db mice, alleviated dyslipidemia, and helped in SM glucose uptake. Protein tyrosine phosphatase 1B (PTP1B) is a major negative regulator of the insulin signaling pathway. The inhibition of this enzyme by GF4 resulted in increased insulin receptor and insulin receptor substrate 1 tyrosine phosphorylation and enhanced insulin sensitivity. Overall, GF4 activates the insulin signaling pathway by inhibiting PTP1B [41]. Further, GF4 has an inhibitory effect on human lymphocytoma Jurkat (JK) cell by inducing apoptosis [42].

## 7. Ginsenoside Ra1 (GRa1)

GRa1 is a key active ingredient in ginseng with immune regulatory, anti-inflammatory, and anti-oxidant properties [43]. Little is known about the effects of GRa1 on human health compared to other known ginseng compounds. GRa1 can affect the cardiovascular system, immune regulation, and nervous system [44]. It is present in both red ginseng powder and red ginseng concentrate samples [45]. GRa1 has been reported as a significant inhibitor of protein tyrosine kinase activation induced by in vitro hypoxia/reoxygenation in cultured human umbilical vein endothelial cells [46]. However, GRa1 has been shown to have anti-inflammatory and anti-oxidant properties, and thus it might be effective for the management of cancers and other inflammation-related diseases such as autoimmune diseases (rheumatoid arthritis), cardiovascular diseases (high blood pressure and heart disease), and gastrointestinal disorders (inflammatory bowel disease).

## 8. Ginsenoside Rb1 (GRb1)

In vitro and in vivo studies have demonstrated GRb1 to have diverse pharmacological applications in metabolic disorders due to its anti-apoptotic effects and ability to regulate oxidative stress, inflammatory responses, and autophagy. In addition, GRb1 suppresses obesity, hyperglycemia, and diabetes by regulating glycolipid metabolism and improving insulin and leptin sensitivities [47]. GRb1 may increase insulin sensitivity by downregulating 11β-hydroxysteroid dehydrogenase type I in T2D [48]. The administration of GRb1 (60 mg/kg of body mass intraperitoneally (i.p.)) daily for 12 days decreased adipose tissue and leptin levels in KK-Ay DM mice [49]. Furthermore, GRb1 (20 mg/kg of body mass, daily) reduced hepatic fat formation and enhanced insulin sensitivity in obese diabetic db/db mice; these effects were confirmed by reductions in liver weight and hepatic triglyceride contents [50]. GRb1 at 10 mg/kg of body mass (i.p.) daily considerably reduced body weight gain, improved glucose tolerance, and increased fasting plasma insulin levels in high-fat diet-induced obese mice and rats [51,52]. In addition, GRb1 increased GLUT4 translocation in C2C12 myotubes and 3T3-L1 cells by activating the adiponectin signaling pathway [53]. Notably, GRb1 is a major component of ginseng [53], which is frequently used as a natural medication in diabetic patients. Altogether, GRb1 has the potential to be used as an anti-obesity, anti-hyperglycemic, and anti-diabetic drug that affects multiple targets.

## 9. Ginsenoside Rb2 (GRb2)

It is a PPD-type saponin abundant in the stems and leaves of ginseng [54]. GRb2 significantly enhanced the viability of HT22 murine hippocampal neuronal cells [55] and inhibited the growth, migration, and invasion of colorectal cancer cells (HT29 and SW620 cell lines) [56]. GRb2 has been used to manage atherosclerosis [57], insulin resistance and obesity [58], endothelial cell senescence [59], and suppression of glutamate-induced neurotoxicity [55]. Thus, GRb2 appears to have potential for treating diabetes, obesity, tumors, viral infections, and cardiovascular conditions.

## 10. Ginsenoside Rb3 (GRb3)

GRb3 is a ginseng-derived natural product with cardioprotective properties [60] that can reduce the risk of myocardial infarction by inhibiting oxidative stress and suppressing inflammation [61]. It was found in an in vivo study to reduce the levels of the inflammatory markers NF-κB and CD45 and enhance the activities of crucial proteins of the contraction unit (cardiac troponin protein I (cTnI) and α-actinin) to recover cardiac function [62]. Furthermore, in vivo and in vitro studies have shown GRb3 mitigates oxidative stress by triggering the anti-oxidation signaling of PERK/Nrf2/HMOX1 [63]. Studies indicate that GRb3 may be helpful for treating heart-related disorders.

## 11. Ginsenoside Rc (GRc)

GRc has been reported to enhance bone development in ovariectomy-induced osteoporotic mice and to stimulate osteogenic differentiation in vitro through the Wnt/β-catenin signaling pathway [64]. Additionally, an in vivo study demonstrated that GRc administration significantly attenuated acetaminophen-induced hepatotoxicity, repaired liver damage, and improved survival [65]. In addition, in a dose-dependent manner, GRc reduced the proliferation and viability of 3T3L1 preadipocytes, adipocyte numbers, and lipid accumulation in maturing 3T3L1 preadipocytes, indicating it inhibited lipogenesis [66]. Collectively, it would appear that GRc has potential utility for managing several diseases.

## 12. Ginsenoside Rd (GRd)

The leading causes of muscle wasting are aging and cancer, and there are no effective cures for these conditions. However, GRd has been shown to alleviate muscle wasting. In mice, GRd administration suppressed age- and cancer-induced muscle atrophy and improved grip strength, hanging times, muscle mass, and muscle tissue cross-sectional areas; at the molecular level, GRd inhibited STAT3 phosphorylation and suppressed atrogin-1, muscle RING-finger protein-1 (MuRF-1), and myostatin levels [67]. Myostatin is a well-known inhibitor of muscle development [68]. MuRF1 is a key factor in the SM atrophy process that occurs during catabolic conditions, making MuRF1 a promising target for pharmaceutical therapies for muscle-wasting conditions [69]. SM improvement is necessary for healthy life [70,71]. Furthermore, GRd improved ischemic stroke-induced damage by suppressing oxidative stress and inflammation, prolonging neural cell survival by upregulating the endogenous anti-oxidant system and phosphoinositide-3-kinase/AKT signaling [72]. Thus, GRd may be an innovative natural product for treating muscle-wasting conditions, act as an anti-diabetic therapy by improving muscle health, and have anti-inflammatory, neuroprotective, and cardioprotective properties.

## 13. Ginsenoside Rf (GRf)

GRf is a constituent of Korean ginseng and upregulates markers of myoblast differentiation and mitochondrial biogenesis. GRf improves exercise tolerance in mice, possibly by enhancing mitochondrial biogenesis and myoblast differentiation via AMPK and p38 MAPK signaling pathways, suggesting that GRf boosts energy production to meet the increased demands of working muscle cells [73]. In addition, GRf has been reported to have neuroprotective and anti-inflammatory effects under hypoxic conditions. The binding of GRf at the active site of PPARγ suggests that it binds at the position used by known agonists [74]. In 3T3L1 adipocytes, GRf treatment downregulated PPARγ and perilipin (lipid droplet-associated protein) levels and reduced lipid accumulation [75]. These observations suggest GRf might be useful for treating obesity.

## 14. Ginsenoside Rg2 (GRg2)

GRg2 promotes porcine mesenchymal stem cell (pMSC) proliferation, prevents D-galactose-induced oxidative stress and senescence, and increases autophagic activity through the AMPK signaling pathway. Furthermore, long-term culture with GRg2 promoted pMSC proliferation, prevented replicative senescence, and preserved stemness [76]. In addition, the ability of GRg2 to exert its anti-atherosclerotic effects at the cellular and animal levels supports ginseng’s role as a functional dietary regulator [77]. Additionally, GRg2 was found to decrease mRNA levels of the inflammatory factors TNF, IL-6, and IL-8, and at 20 μM, it considerably suppressed IL-6, IL-8, and IL-1 [77]. These findings suggest a strategy for muscle regeneration based on the in vitro expansion of pMSCs.

## 15. Ginsenoside Rg3 (GRg3)

Acute pancreatitis (AP) is a systemic inflammatory response syndrome. In a study performed using a cerulein-induced murine model of AP to investigate the effect of GRg3, cerulein increased serum amylase, TNFα, IL-6, IL-1β, ROS, and Fe^2+^ levels, and GRg3 co-treatment decreased cerulein-induced ROS buildup and cell death in pancreatic tissues [78]. Due to the lack of an effective delivery approach, it is difficult to deliver GRg3 to body organs due to its hydrophobic nature. However, the intramyocardial injection of GRg3-loaded PEG-b-PPS nanoparticles in a rat ischemia–reperfusion model improved cardiac functions and reduced infarct sizes [79].

## 16. Ginsenoside Rg5 (GRg5)

GRg5 was administered to nude mice bearing A549/T tumors to combat multidrug resistance. Treatment with GRg5 and docetaxel considerably suppressed the growth of drug-resistant tumors without increasing toxicity compared to docetaxel alone at the same dose [80]. In another study, GRg5 remarkably suppressed breast cancer cell propagation by inducing mitochondria-mediated apoptosis and autophagic cell death. It was also found that GRg5 decreased the phosphorylation of PI3K, Akt, and mTOR and attenuated PI3K/Akt signaling in breast cancer [81]. Thus, GRg5 has therapeutic potential as a breast cancer treatment. Furthermore, GRg5 [82] and GRh1 [83] can both alleviate cisplatin-induced nephrotoxicity [82], presumably due to their anti-oxidant, anti-apoptotic, and anti-inflammatory effects.

## 17. Ginsenoside Rh1 (GRh1)

GRh1 is obtained from red ginseng and used to improve physical fitness. In a cisplatin-induced injury model, GRh1 enhanced the vitality of HK-2 cells and inhibited ROS production and apoptosis [83], which suggested that GRh1 has potential use for alleviating cisplatin-induced nephrotoxicity in cancer patients. In addition, GRh1 was found to have an anti-cancer effect on breast cancer by inhibiting the ROS-mediated PI3K/Akt pathway and causing cell cycle arrest, apoptosis, and autophagy [84]. An in vitro study reported that GRh1 (at 100 µM) significantly inhibited cell migration and invasion and effectively inhibited colorectal cancer development [85]. Currently, GRh1 is mostly being used as an anticancer agent and could be further explored for its use against various other cancers.

## 18. Ginsenoside Rh2 (GRh2)

GRh2 is obtained from the roots of *P. ginseng* and has been reported to have anti-tumor effects by immunomodulating the tumor microenvironment (TME) [86], regulating HMGB1/NF-κB signaling, and improving the oxygen–glucose deprived environment of cardiomyocytes [87]. However, the in vivo effects of GRh2 have not yet been well explored, and its therapeutic effects are unknown.

## 19. Ginsenoside Rh3 (GRh3)

Lung cancer is the second most common cause of cancer-related death after breast cancer [88]. In vitro, GRh3 (at 50 μM) inhibited the proliferation of A549 and PC9 cells, and in another in vitro study, GRh3 inhibited tumor growth by causing cell arrest in the G1 phase. In vivo, GRh3 at 50 and 100 mg/kg significantly inhibited lung cancer metastasis [89]. GRh3 also inhibited HCT116 (colon cancer) cell proliferation, invasion, migration, and arrested cells in the G1 phase by downregulating genes related to DNA replication [90]. In addition, GRh3 significantly ameliorated myocardial necrosis and caspase 3 levels in male Sprague Dawley rat myocardial tissues by hindering the p38 MAPK pathway [91].

## 20. Ginsenoside Rh4 (GRh4)

GRh4 significantly inhibited the production of pro-inflammatory cytokines (TNF-α, IL-6, and IL-1β) in RAW264.7 cells (macrophages). JAK-STAT, TNF, NF-κB, and PI3K-Akt were identified as the main pathways used by GRh4 to reduce inflammation [92]. It has also been reported that GRh4 protects kidneys from cisplatin-induced oxidative injury [93]. GRh4 has been shown to have a high anti-lung adenocarcinoma efficacy in vitro and in vivo. Lung adenocarcinoma is a typical cellular breakdown in the lungs with a high harm that desperately should be treated [94]. Further, it is reported that GRh4 inhibits colorectal cancer cell proliferation [95], breast cancer growth [96], and delays SM aging through the SIRT1 pathway [97].

## 21. Ginsenoside Rh7 (GRh7)

GRh7 has been reported to inhibit H1299 (a lymph node-derived human non-small cell lung cancer cell line) growth (by 83%) and proliferation. In the same study, A549 (also an NSCLC cell line) and H1299 cells were used to study the time-dependent effects of GRh7 on cell growth. After treatment with 25 μM GRh7 for 4 days, A549 growth was inhibited by 72% and H1299 growth by 75% compared to non-treated controls [98]. These results suggest GRh7 has anti-cancerous properties that warrant further investigation.

## 22. Ginsenoside Rk1 (GRk1)

GRk1 is produced by thermally dehydrating GRg3, a saponin present in *Panax ginseng* Meyer. GRk1 effectively inhibited *N*-methyl-d-aspartate receptors in cultured hippocampal neurons [99] and protected human melanocytes from H_2_O_2_-induced death. PIG1 melanocytes were pretreated with GRk1 at 0, 0.1, 0.2, or 0.4 mM for 2 h and then exposed to H_2_O_2_ under cytotoxic conditions (at 1.0 mM for 24 h). GRk1 pretreatment at 0.2 or 0.4 mM for 2 h considerably improved cell viability and decreased cell shrinkage versus H_2_O_2_ treatment controls [100]. In another study, GRk1 treatment reversed cisplatin-induced increases in the protein levels of Bax, cleaved caspase 3 and 9, and Bcl-2 [101]. GRk1 was also reported to have anti-tumor activity against lung squamous cell carcinoma [102], and at 30 mg/kg, GRk1 injections markedly inhibited tumor xenograft growth [103].

## 23. Ginsenoside Rk3 (GRk3)

GRk3 is a key bioactive constituent in ginseng and has robust anti-oxidant properties. GRk3 was found to enhance neuronal apoptosis, decrease intracellular ROS production, and restore mitochondrial membrane potentials in PC12 and primary neuronal cells. GRk3 also improved spatial learning and reduced memory deficits in an amyloid precursor protein (APP)/presenilin 1 (PS1) double transgenic mouse model of Alzheimer’s disease (AD) [104]. AD has devastating effects on society with a limited number of approaches for its treatment [105]. AD is caused by mutations in one of the genes that codes for APP and presenilins 1 and 2. The majority of these gene mutations boost Aβ42 production [106]. Parkinson’s disease (PD) is the second most common ND without any proper cure [107] and is categorized as a movement disorder. Natural products for the management of PD have also been reported [108]. Additionally, several other natural products have been reported for the management of AD and other NDs [109,110]. The anti-cancer effects of GRk3 were also checked in Eca109 and KYSE150 cell lines (both esophageal squamous carcinoma cell lines), and it was observed that GRk3 suppressed proliferation and colony formation for both cell types. This inhibition was ascribed to blocking of the PI3K/Akt/mTOR pathway and consequent activation of apoptosis and autophagy [111]. GRk3 might be an effective anti-tumor agent for esophageal cancer and for the treatment of renal dysfunctions caused by cisplatin-induced oxidative injury [93]. Furthermore, GRk3 improved hematopoietic function in myelosuppressed mice [112] and inhibited the proliferation, migration, and invasion associated with the extramedullary infiltration of leukemia [113].

## 24. Ginsenoside Ro (GRo)

GRo is a primary saponin in *P. ginseng* C.A. Meyer with several biological actions. In B16F10 tumor-bearing mice, GRo significantly inhibited tumor growth [114], LPS-induced lung damage, and TNF, IL-6, and IL-1 transcript levels in tumor tissues. Furthermore, in a dose-dependent manner, GRo suppressed the phosphorylation of NF-κB and MAPKs and the nuclear translocation of the p65 subunit. These findings imply that GRo targets inflammation by directly inhibiting the TLR4 signaling pathway [115]. The anti-inflammatory effects of GRo have been linked to a significant decrease in levels of pro-inflammatory cytokines generated by lipopolysaccharides. In a dose-dependent manner, GRo enhances cell survival while lowering reactive oxygen species (ROS) and nitric oxide generation produced by lipopolysaccharides [116]. Further, GRo improves obesity and insulin resistance in mice [117].

## 25. Floralginsenoside A (FGA)

Melanin provides UV protection and removes ROS from the skin. However, excessive melanin production and its accumulation in the skin can result in pigmentation disorders (e.g., solar lentigo, melasma, and freckles) [118]. Melan-a cells, an immortalized C57BL/6 mouse melanocyte cell line containing high levels of melanin, were used to investigate the melanin-inhibitory activity of FGA. At a concentration of 160 μM, FGA inhibited melanin activity by 23.9% without causing cytotoxicity. Tyrosinase is a key player in the biosynthesis of melanin, and the main mechanism underlying the anti-melanogenesis effects of inhibitory agents involves the downregulation of MITF (microphthalmia-related transcription factor) due to the ERK-induced phosphorylation of MITF at serine-73, which triggers the ubiquitination of MITF and its subsequent degradation. The phosphorylation of MITF at serine 29 activates Akt signaling and inhibits melanin production. FGA can block MITF–tyrosinase signaling and/or activate ERK–Akt signaling, which are both involved in melanogenesis. In addition, FGA treatment dose-dependently decreases the expressions of tyrosinase and MITF. Furthermore, FGA (at 160 μM) significantly and dose-dependently augmented phospho-ERK and Akt signaling pathways [119]. Tyrosinase activity and melanin content inhibition may result in skin whitening. However, the potential carcinogenic side effects of the agent currently used (kojic acid) to whiten skin [120] necessitates the development of new, safer, more effective depigmenting agents, and natural products feature prominently in these studies. Makeup/cosmetic production is expanding, especially in South Korea. Thus, the identification of natural makeup agents is likely to result in commercially attractive health and skin care products.

## 26. Future Perspectives

This study was performed to summarize what is known about the influence of ginseng and its derived natural products on diseases. It is well known that synthetic drugs are associated with adverse effects and that herbal remedies have been used for millennia and are relatively free of side effects. As a result, perceptions are changing in favor of natural therapies and traditional medicines. In particular, ginseng has been administered as an herbal medicine for thousands of years and is now commercially available in pill and tea forms. Intriguingly, one clinical study reported that patients who took ginseng after curative surgery had a 38% higher overall survival rate and a 35% higher 5-year disease-free rate [19,121].

The roles and functions of a number of ginseng compounds are listed in Table 2. Several of the natural products isolated from ginseng are therapeutically beneficial. Natural products have been thoroughly investigated in vitro and in vivo, and we suggest in silico studies be undertaken to aid in the primary screening of ginseng natural products. It has been reported that in silico-screened compounds produce better results during subsequent in vitro or in vivo testing [122,123,124]. Most of the natural products mentioned have anti-diabetic, anti-neuroprotective, anti-cancer, anti-oxidant, and anti-inflammatory effects. We suggest that the repurposing of these compounds be attempted to improve their therapeutic effects.

## 27. Conclusions

Ginseng and ginseng-derived natural products are attractive candidates for the treatment of several disease. Furthermore, this study showed that ginseng and its derived natural products are powerful therapeutic agents/supplements that improve health and increase energy. Therefore, we suggest that clinical trials be performed to confirm the therapeutic efficacies of ginsenosides in cancer, stroke, obesity, aging, and NDs.

## Figures and Tables

**Table 1:** List of several known compounds of ginseng along with their molecular formula and weight. The compounds known in the different parts of the ginseng plant, such as root and flower bud, having higher molecular weight as compared to other parts of the ginseng plant.

Part of Ginseng	Compounds Name	PubChem ID	Molecular Formula	Molecular Weight (g/mol)
Hydrolysis	Protopanaxadiol (PPD)	9920281	C_30_H_52_O_3_	460.7
	Protopanaxatriol (PPT)	9847853	C_30_H_52_O_4_	476.7
	Panaxadiol	73498	C_30_H_52_O_3_	460.7
	Panaxatriol	73599	C_30_H_52_O_4_	476.7
Leaves	Ginsenoside F1	9809542	C_36_H_62_O_9_	638.9
	Ginsenoside F2	9918692	C_42_H_72_O_13_	785.0
	Ginsenoside F3	46887678	C_41_H_70_O_13_	771.0
	Ginsenoside F4	102004835	C_42_H_70_O_12_	767.0
	Ginsenoside Ki	102294899	C_37_H_64_O_10_	668.9
	Ginsenoside Km	102294900	C_37_H_64_O_10_	668.9
	Ginsenoside Rh6	131752646	C_36_H_62_O_11_	670.9
	Ginsenoside Rh7	101096472	C_36_H_60_O9	636.9
	Ginsenoside Rh8	85245726	C_36_H_60_O_9_	636.9
Roots	Ginsenoside Ra1	100941542	C_58_H_98_O_26_	1211.4
	Ginsenoside Ra2	100941543	C_58_H_98_O_26_	1211.4
	Ginsenoside Ra3	73157064	C_59_H_100_O_27_	1241.4
	Ginsenoside Rb1	9898279	C_54_H_92_O_23_	1109.3
	Malonylginsenoside Rb1	118987129	C_57_H_94_O_26_	1195.3
	Ginsenoside Rb2	6917976	C_53_H_90_O_22_	1079.3
	Ginsenoside Rb3	12912363	C_53_H_90_O_22_	1079.3
	Ginsenoside Rc	12855889	C_53_H_90_O_22_	1079.3
	Ginsenoside Rd	11679800	C_48_H_82_O_18_	947.2
	Ginsenoside Rf	441922	C_42_H_72_O_14_	801.0
	20-Glucoginsenoside Rf	3052077	C_48_H_82_O_19_	963.2
	Ginsenoside Rg1	441923	C_42_H_72_O_14_	801.0
	Ginsenoside Rg2	21599924	C_42_H_72_O_13_	785.0
	Ginsenoside Ro	11815492	C_48_H_76_O_19_	957.1
	Ginsenoside Rs1	85044013	C_55_H_92_O_23_	1121.3
	Ginsenoside Rs2	162343294	C_55_H_92_O_23_	1121.3
Steamed roots	Ginsenoside Rg3	9918693	C_42_H_72_O_13_	785.0
	Ginsenoside Rg5	11550001	C_42_H_70_O_12_	767.0
	Ginsenoside Rg6	91895489	C_42_H_70_O_12_	767.0
	Ginsenoside Rh1	12855920	C_36_H_62_O_9_	638.9
	Ginsenoside Rh2	119307	C_36_H_62_O_8_	622.9
	Ginsenoside Rh3	20839223	C_36_H_60_O_7_	604.9
	Ginsenoside Rh4	21599928	C_36_H_60_O_8_	620.9
	Ginsenoside Rh5	10699455	C_37_H_64_O_9_	652.9
	Ginsenoside Rk1	11499198	C_42_H_70_O_12_	767.0
	Ginsenoside Rk2	90472238	C_36_H_60_O_7_	604.9
	Ginsenoside Rk3	75412555	C_36_H_60_O_8_	620.9
	Ginsenoside Rs3	100937823	C_44_H_74_O_14_	827.0
	Ginsenoside Rs5	102021585	C_44_H_72_O_13_	809.0
Flower buds	Floralginsenoside A	16655581	C_42_H_72_O_16_	833.0
	Floralginsenoside B	101423532	C_50_H_84_O_21_	1021.2
	Floralginsenoside C	16655212	C_42_H_72_O_15_	817.0
	Floralginsenoside D	16655213	C_42_H_72_O_15_	817.0
	Floralginsenoside E	101423533	C_41_H_70_O_15_	803.0
	Floralginsenoside F	101423534	C_48_H_82_O_20_	979.2
	Floralginsenoside G	101423535	C_48_H_82_O_21_	995.2
	Floralginsenoside H	101423536	C_53_H_90_O_25_	1127.3
	Floralginsenoside I	16655580	C_42_H_72_O_16_	833.0
	Floralginsenoside J	101423537	C_41_H_70_O_15_	803.0
	Floralginsenoside K	101423538	C_50_H_84_O_21_	1021.2
	Floralginsenoside Lb	102512867	C_48_H_82_O_19_	963.2
	Floralginsenoside M	101423540	C_48_H_82_O_19_	963.2
	Floralginsenoside N	101423541	C_53_H_90_O_22_	1079.3
	Floralginsenoside O	101423542	C_53_H_90_O_22_	1079.3
	Floralginsenoside P	101423543	C_53_H_90_O_23_	1095.3
	Floralginsenoside Ta	46224641	C_36_H_60_O_10_	652.9
	Floralginsenoside Tb	46224642	C_35_H_62_O_11_	658.9
	Floralginsenoside Tc	46224643	C_53_H_90_O_25_	1127.3
	Floralginsenoside Td	46224646	C_53_H_90_O_25_	1127.3
	Ginsenoside I	102050355	C_48_H_82_O_20_	979.2
	Ginsenoside II	101717751	C_48_H_82_O_19_	963.2
Fruits	25-Hydroxyprotopanaxadiol	158501	C_30_H_54_O_4_	478.7
Seeds	Panaxadione	25233029	C_30_H_48_O_5_	488.7

**Table 2:** Therapeutic application of different ginseng compounds in disease management.

Compound Name	Function	Model/Object/Experiments	Reference
Protopanaxadiol	recovery from endometriosis	mice	[29]
Ginsenoside F2	alcoholic liver damage improvement	C57BL/6J WT or IL-10 knockout mice	[37]
Ginsenoside F1	repair the vascular defects caused by axitinib in zebrafish	in vivo tests in zebrafish	[33]
	reduce Aβ-induced cytotoxicity	neuroblastoma neuro-2a (mouse) and neuroblastoma SH-SY5Y (human)	[34]
Ginsenoside Rh7	anticancerous properties	A549 and H1299 cell line	[98]
Ginsenoside Rb1	decrease adipose tissue and leptin levels	KK-Ay DM mice	[49]
	reduce hepatic fat formation	obese diabetic db/db mice	[50]
	reduce body weight gain	HFD-induced obese mice	[51]
	increase GLUT4 translocation	C2C12 and 3T3-L1 cells	[53]
Ginsenoside Rb2	improve cell viability	HT22 murine hippocampal neuronal cells	[55]
	inhibit the growth of colorectal cancer cells	HT29 and SW620 cell lines	[56]
Ginsenoside Rc	enhance bone development	ovariectomy-induced osteoporosis mice	[64]
	reduce the proliferation and viability process	3T3L1	[66]
Ginsenoside Rd	enhance hypertrophy	aged mice	[67]
Ginsenoside Rg2	encourage pMSC proliferation	MTT assay	[76]
Ginsenoside Ro	inhibit tumor growth	B16F10 tumor-bearing mice	[114]
Ginsenoside Rg3	decrease ROS buildup	mice	[78]
Ginsenoside Rh2	improve the oxygen–glucose deprivation environment of cardiomyocytes	regulate the HMGB1/NF-κB signaling	[87]
Ginsenoside Rh1	anticancer effect on breast cancer cells	inhibition of the ROS-mediated PI3K/Akt pathway	[84]
Ginsenoside Rh3	inhibit proliferation	A549 and PC9 cells	[89]
Ginsenoside Rk3	improve neuronal apoptosis	PC12 and primary neuronal cells	[104]
Floralginsenoside A	melanin inhibitory activity	C57BL/6 mouse melanocyte cell line	[119]

## Data Availability

Not applicable.
